# MS‐275 potentiates the effect of YM‐155 in lung adenocarcinoma via survivin downregulation induced by miR‐138 and miR‐195

**DOI:** 10.1111/1759-7714.13076

**Published:** 2019-05-14

**Authors:** Bai‐Ling Luo, Yan Zhou, Hui Lv, Sheng‐Hua Sun, Wen‐Xiang Tang

**Affiliations:** ^1^ Respiratory Department The First Xiangya Hospital of Central South University Changsha China; ^2^ Department of Pathology, School of Medicine University of Colorado Anschutz Medical Campus Aurora, Colorado USA; ^3^ Respiratory Department The Third Xiangya Hospital of Central South University Changsha China

**Keywords:** LUAD, MS‐275, NSCLC, survivin, YM‐155

## Abstract

**Background:**

YM‐155 has been proven to be an efficient antitumor suppressor in non‐small cell lung cancer (NSCLC) cells. However, the suppressive effect of YM‐155 on the expression of survivin is not sufficient and has a short half‐life. MS‐275, a histone deacetylase inhibitor, has significant antitumor capacity with a relatively long half‐life. Our study explored whether MS‐275 could enhance the inhibitory effect of YM‐155 on LUAD proliferation.

**Methods:**

To investigate the synergistic effect of MS‐275 and YM‐155, we employed methyl thiazolyl tetrazolium and colony formation assays to access the inhibition effect of MS‐275, YM‐155, or a combination in A549 and HCC827 cell lines. We then detected the effect of MS‐275 and YM‐155 on the expression of survivin and pro‐apoptotic proteins by Western blot and miR‐138 or miR‐195 expression by quantitative PCR. We also analyzed the methylation level of microRNAs (miRNAs) using methylation‐sensitive quantitative PCR. Finally, we investigated the interaction between miRNAs and survivin by luciferase reporter assay.

**Results:**

MS‐275 facilitated an inhibitory effect of YM‐155 on lung adenocarcinoma cell proliferation. MS‐275 can upregulate the level of acetylated H3, promote the degradation of DNA methyltransferases, and inhibit the methylation of miR‐138 and miR‐195 genes to elevate the expression of miR‐138 and miR‐195. Moreover, miR‐138 and miR‐195 showed a synergistic effect with YM‐155 by directly binding to the 3 untranslated region of survivin to attenuate its expression.

**Conclusion:**

For the first time, we report the synergistic effective of MS‐275 and YM‐155 and suggest a new direction for the future application of YM‐155.

## Introduction

Lung adenocarcinoma (LUAD) is the most prominent histological subtype of non‐small cell lung cancer (NSCLC), with high rates of mortality and metastasis. Although some targeted drugs, such as AZD9291^1^ and ceritinib,^2^ have shown benefits on associated *EGFR* gene mutations and *EML4‐ALK* gene rearrangement, respectively, the prognosis of patients with LUAD remains unfavorable, with a five‐year survival rate of only 15%.^3^ Single administrations are often defeated by adverse phenomena, such as inefficacy in clinical experiments or drug resistance.^4^, ^5^ Research is focused on developing new strategies for targeted therapeutics against LUAD progression.

Survivin is a representative member of the inhibitor of apoptosis protein (IAP) family and high expression of survivin has been correlated with poor prognosis and drug resistance among NSCLC patients.^6^ YM‐155, a novel survivin inhibitor, has been used in clinical trials.^7^ YM‐155 can make NSCLC cells sensitive to radiation therapy both in vitro and in vivo, which is likely a result of the inhibition effect of YM‐155 on DNA repair.^8^ It has been reported that YM‐155 also inhibits the transcription of survivin with a slight effect on the expression level of other members of the IAP family by disrupting promoter‐specific transcription factor 1 (Sp1) binding within the −149 to −71 region in the core survivin promoter .^9^ Therefore, survivin has attracted interest as a probable molecular target for cancer therapy. However, with a short half‐life, YM‐155 does not have sufficient inhibition ability against survivin, leading to limitations in clinical practice.^10^

Histone deacetylase (HDAC) inhibitors specifically act on the regulation of histone acetylation, and were the first to be approved as a result of clinical breakthroughs in the treatment of various subtypes of hematological tumors.^11^ As a successful example of a modified molecular‐targeted drug, MS‐275 has high inhibitory efficiency on HDAC1 and HDAC3, with half maximal concentrations of approximately 0.51 μM and 1.7 μM, respectively.^12^ The inhibition effect of MS‐275 has been reported in a variety of tumors, such as human leukemia^13^ and NSCLC.^14^ It has been reported that HDAC inhibitors can decrease antiapoptotic proteins, such as XIAP.^15^ The inhibition effect of MS‐275 on survivin has also been reported.^13^ In addition, MS‐275 is noted for its potent anticancer ability with a long serum half‐life,^16^ whereas YM‐155 has a short half‐life.^10^ Inhibition of HDACs is reported to downregulate the expression of DNA methyltransferase 1 (DNMT1), which is generally known as an inhibitor of tumor suppressive genes via hypermethylation.^17^ MS‐275 is reported to upregulate the expression of antitumor microRNAs (miRNAs) by attenuating the DNMT1 level, thus restraining the downstream oncogenic targets of these miRNAs.^6^ Based on the results of previous studies, the strategy of a combination of YM155 and MS‐275 may potentially overcome the insufficiency of YM‐155 in NSCLC, especially in LUAD.

In the present study, we investigated whether the combination of YM‐155 and MS‐275 induced a significant antitumor effect in A549 and HCC278 cell lines compared to that induced by the administration of either agent alone. We then explored whether the synergistic effect was relative to the level of acetylation H3 and the expression of DNMT1. We determined the combination effect of miR‐138 and miR‐195 mimic treatment with YM‐155 and investigated how it interacted with survivin.

## Methods

### Cell lines and cell culture

The A549 human lung carcinoma epithelial‐like cell line (^#^CCL‐185) and the HCC827 lung adenocarcinoma cell line (^#^CRL‐2868) were obtained from American Type Culture Collection (Rockville, MD, USA). A549 was cultured in Dulbecco's modified Eagle medium added with 10% heat‐inactivated fetal bovine serum, l‐alanyl–l‐glutamine (2 mM), penicillin (100 μg/ml), and streptomycin (100 U/ml). HCC827 was cultured in RPMI‐1640 supplemented with 10% heat‐inactivated fetal bovine serum, penicillin (100 μg/ml), and streptomycin (100 U/ml). Cells were maintained in a humidified atmosphere at 37°C and 5% CO_2_.

### Reagents and antibodies

MS‐275 and YM‐155 were purchased from ChemieTek (Indianapolis, IN, USA). Bovine serum albumin, methyl thiazolyl tetrazolium (MTT), and crystal violet were purchased from Sigma‐Aldrich (St. Louis, MO, USA). The One Step PrimeScript miRNA cDNA Synthesis Kit and SYBR Premix ExTaq II were purchased from TaKaRa Biotechnology (Dalian, China). The Dual‐Luciferase Reporter Assay System was purchased from Promega (Madison, WI, USA). The following primary antibodies were used: cleaved PARP (#5625), cleaved caspase‐3 (#9661), survivin (#2808), Mcl‐1 (#94296), DNMT1 (#5032), acetyl histone 3 (#9649), histone 3 (#4499), and glyceraldehyde 3‐phosphate dehydrogenase (GAPDH, #5174; Cell Signaling Technology, Danvers, MA, USA). The dilutions of all primary antibodies were performed at 1:1000. Secondary antibodies conjugated to horseradish peroxidase (HRP) were purchased from Santa Cruz Biotechnology (Santa Cruz, CA, USA), used at 1:2000 dilutions, and visualized by enhanced chemiluminescence (Cell Signaling Technology). MiR‐138 and miR‐195 mimics and negative control (NC) miRNA were synthesized by GenePharma (Shanghai, China).

### Methyl thiazolyl tetrazolium assay

Cells (5 x 10^3^/well) were seeded in 96‐well plates in 100 μL of growth medium and cultured for two days with different treatments. After the treatment interval, supernatants were discarded and 100 μl 3‐(4,5‐dimethyl‐2‐thiazolyl)‐2,5‐diphenyl‐2‐H‐tetrazolium bromide reagent (0.5 mg/mL) diluted by culture medium was added, followed by incubation for four hours. Dimethyl sulfoxide (100 μl/well) was then added to terminate the reaction. Absorbance was determined at 490 nm in triplicate using a colorimetric plate reader (Thermo Fisher Scientific, Waltham, MA, USA).

### Colony formation assay

Colony formation assay was performed as described previously, with little modification.^18^ Cells were seeded in 24‐well plates at 500 cells/well. Cells were untreated or treated with MS‐275, YM‐155, miR‐138 mimic, miR‐195 mimic, or a combination for 48 hours. After rinsing with fresh medium, cells were incubated for 14 days to form colonies, stained with crystal violet (0.4 g/L), and scored using a microscope. Colonies were only calculated if a single clone included > 100 cells. Each assay was performed in triplicate on two independent occasions. Colony assay was used to elucidate the possible differences in long‐term effects on human A549 or HCC287 cells.

### Xenograft tumorigenesis

All animal experiments were undertaken in accordance with the Care and Use of Laboratory Animals guidelines of Central South University. A549 cells (5 x 10^5^/mL) were injected into the flanks of six week old male BALB/c nu/nu mice purchased from SLRC Experimental Animal Co. Ltd. (Shanghai, China). After two weeks, when the tumors reached an approximate average volume of 50 mm^3^, the mice were randomized into four groups (5 mice/group). The control group received 1% polysorbate resuspended in deionized water. The remaining three groups were treated with YM155 (5 mg/kg body weight), MS‐275 (20 mg/kg body weight), or YM‐155 plus MS‐275 intraperitoneally for three days over four weeks. The tumors were measured every five days. At the indicated time, the mice were sacrificed and the tumor volumes were calculated using the following formula:

$$\text{Volume}=\text{length}\;x\;{\text{width}}^{2}/2.$$

### Western blot analysis

A549 and HCC278 cells were washed with cold phosphate buffered saline, harvested, and then lysed in radioimmunoprecipitation assay lysis buffer for 15 minutes on ice. Samples were centrifuged at 12 000 g for 15 minutes, supernatants were isolated, and the protein was quantified using a Protein Assay Kit (Thermo Fisher Scientific). Equal amounts of protein were separated by 12% sodium dodecyl sulfate‐polyacrylamide gel electrophoresis and then electro‐transferred onto polyvinylidene fluoride membrane. Non‐specific antibody binding was blocked using 4% bovine serum albumin dissolved in 0.1% tris‐buffered saline plus Tween 20 (TBST). The polyvinylidene fluoride membranes were then incubated with appropriate dilutions of primary antibody overnight at 4°C. The antibody‐labeled blots were washed three times with TBST and then incubated with a 1:2000 dilution of HRP‐conjugated secondary antibody in TBST at room temperature for one hour. The secondary HRP‐conjugated antibody was detected by using an electrochemiluminescence detection system (Amersham, Biosciences, Buckinghamshire, UK). Bands were quantified using Quantity One version 4.6.2.

### Quantitative real‐time PCR

Total RNA was extracted using TRIzol according to the manufacturer's instructions. Reverse transcription and quantitative PCR were performed using the One Step PrimeScript miRNA cDNA Synthesis Kit and SYBR Premix Ex Taq II using the ABI 7500 Real Time PCR system (Applied Biosystems, Foster City, CA, USA). Triplicate samples containing complementary DNA were prepared as mentioned above. The *U6* gene and GAPDH was used as an endogenous control for normalization. All real‐time PCR reactions were performed in triplicate, and relative quantifications were calculated using the2^‐ΔΔCt^ method. The PCR conditions used for miRNA quantification were as follows: 95°C for 10 minutes, followed by 45 cycles of 95°C for 15 seconds, 60°C for 1 minute, and a dissociation stage. The primer pairs were as follows:

Survivin‐F: CATTCGTCCGGTTGCGC; Survivin‐R: GATGGCACGGCGCACTTTC; hsa‐miR‐138‐5p‐F: CCCAGCTGGTGTTGTGAATC; hsa‐miR‐138‐5p‐R: CGGCCCAGTGTTCAGACTAC; hsa‐miR‐195‐5p‐F: TGCGCTAGCAGCACAGAAAT; hsa‐miR‐195‐5p‐R: CGGCCCAGTGTTCAGACTAC; GAPDH‐F: CCAGGTGGTCTCCTCTGA; GAPDH‐R: GCTGTAGCCAAATCGTTGT; U6‐F: CTCGCTTCGGCAGCACA; U6‐R: AACGCTTCACGAATTTGCGT.

### Methylation‐specific PCR

Cells (5 x 10^5^/mL) were seeded and incubated with 2 or 5 μM MS‐275 for 48 hours. After treatment with MS‐275, cells were placed in fresh medium and harvested on day 8. Genomic DNA was extracted from the cells, followed by bisulfite treatment using the CpG DNA Modification Kit (Millipore, Billerica, MA, USA) according to the manufacturer's instructions. After chemical modification of the DNA, PCR analysis was performed using primers that were designed specifically to utilize the sequence differences between the methylated and unmethylated DNA resulting from bisulfate treatment. Control DNA was used to determine the quality of bisulfite modification. CpGenome Universal Methylated DNA and Unmethylated DNA were used as positive and negative controls, respectively. All presented data reached preset acceptance criteria.

### Luciferase reporter assay

The sequences of wild‐type 3’ untranslated region (UTR) of survivin were cloned into the downstream of the luciferase gene in the pGL‐3 basic vector (Promega). After A549 and HCC278 cells were co‐transfected with 3’‐UTR reporter plasmid, 20 ng pRL‐TK vector (Promega), and 20 nmol/L of miR‐138 or miR‐195 mimics or NC, respectively, the luciferase activities were measured at the indicated time point using the Dual‐Luciferase Reporter Assay System (Promega). The regulatory effect of miRNAs on survivin was assessed by dividing the results of normalized firefly luciferase activity with the results obtained from Renilla luciferase.

### Transfection

The miR‐138 and miR‐195 mimics, as well as their respective NCs, were synthetized by GenePharma for in vitro transfection. A549 and HCC827 cells were transiently transfected with 50 nmol/L miR‐138 and miR‐195 mimics or miRNA control using Lipofectamine 2000 according to the manufacturer's protocol (Invitrogen, Carlsbad, CA, USA). Transfected cells were incubated for 24 hours at 37°C, followed by biological assays.

### Statistical analysis

Data are shown as mean values ± standard deviation. One‐way analysis of variance followed by Tukey's post hoc comparison test was used to make comparisons among multiple groups. A *P* value of ˂ 0.05 was considered statistically significant.

## Results

### MS‐275 and YM‐155 inhibited lung adenocarcinoma (LUAD) cell proliferation

**Figure 1:**
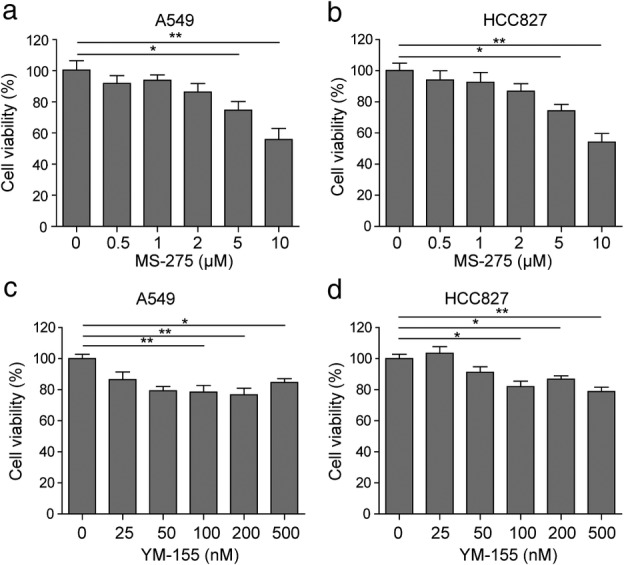
MS‐275 and YM‐155 inhibits lung adenocarcinoma (LUAD) cell proliferation. Methyl thiazolyl tetrazolium assay results of LUAD cell lines A549 and HCC827 following treatment with serial (**a,b**) MS‐275 and (**c,d**) YM155 dilutions for 48 hours. *P < 0.05, **P < 0.01. Data are presented as mean ± standard deviation. At least three independent experiments were performed.

First, the antiproliferative activity of MS‐275 or YM155 against LUAD cell lines A549 and HCC827 was examined. As shown in Figure 1a,b, the inhibitory effect of MS‐275 on cell proliferation enhanced with the increased dose. Particularly, 5 μM and 10 μM MS‐275 had a significant inhibitory effect on the proliferation of A549 (74.73 ± 5.52 for 5 μM, 0.55.92 ± 7.03 for 10 μM) and HCC827 (74.22 ± 4.17 for 5 μM, 54.25 ± 5.52 for 10 μM) cell lines. As shown in Figure 1c,d, YM155 significantly repressed cell proliferation at concentrations of 100, 200, and 500 nM. These cell lines were also sensitive to YM155 treatment, with approximately 20% inhibition concentrations of 200 nM in A549 (76.77 ± 4.22) and HCC827 (86.82 ± 2.12) cell lines.

### A combination of MS‐275 and YM‐155 enhances the inhibitory effect of YM‐155 on proliferation and xenograft tumorigenesis of LUAD cells

**Figure 2:**
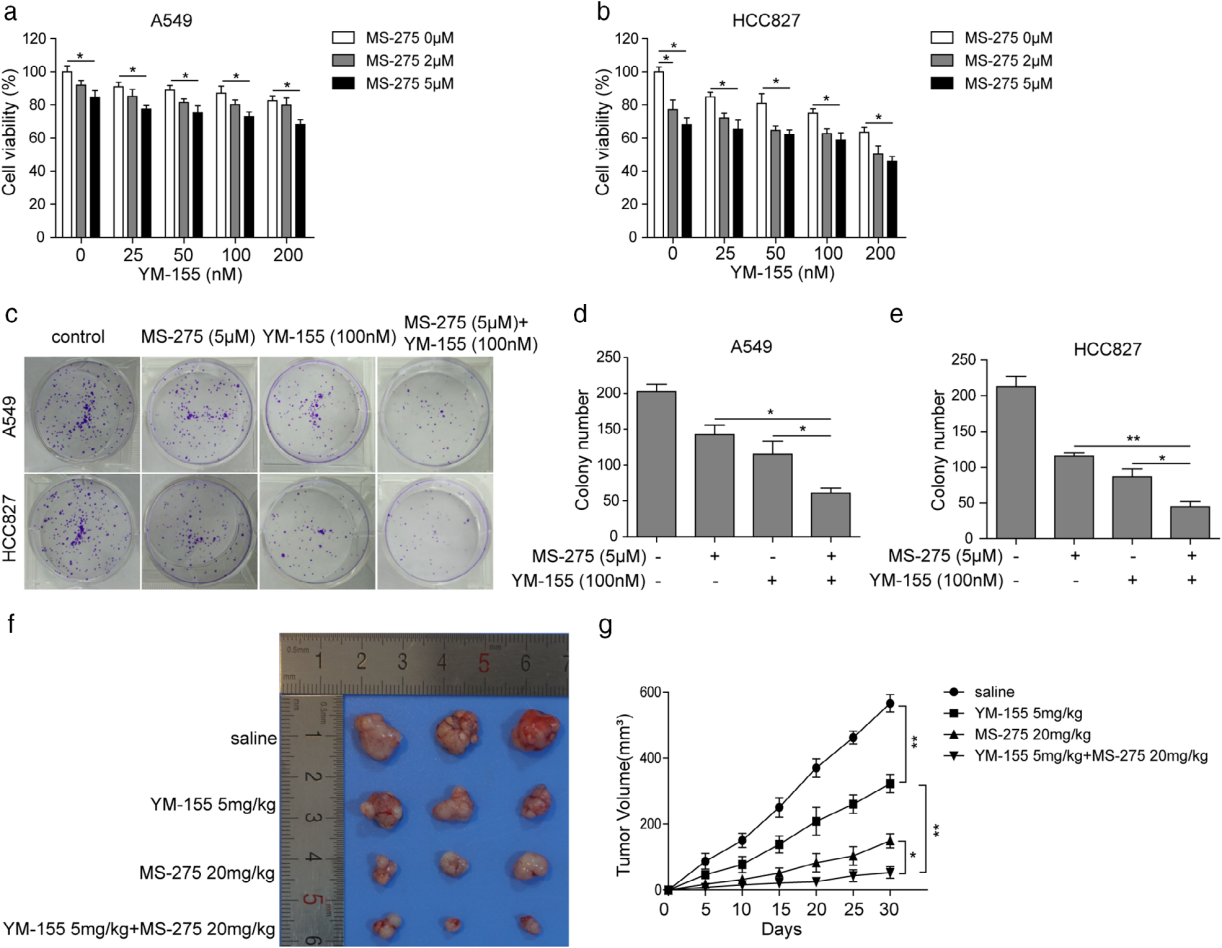
The combination of MS‐275 and YM‐155 enhances the inhibitory effect of YM‐155 on proliferation and xenograft tumorigenesis of lung adenocarcinoma (LUAD) cells. (**a,b**) Methyl thiazolyl tetrazolium (MTT) assay results of LUAD cell lines A549 and HCC827 following treatment with a combination of MS‐275 and YM‐155 for 48 hours. (**c–e**) Colony formation assay results of LUAD cell lines A549 and HCC827 following treatment with 5 μM MS‐275, 100 nM YM‐155, or both for 48 hours. (**f,g**) Xenograft tumor assay results of LUAD cell line A549 following treatment with 20 mg/kg MS‐275, 5 mg/kg YM‐155, or both for 30 days. *P < 0.05, **P < 0.01. Data are presented as mean ± standard deviation. At least three independent experiments were performed.

**Figure 3:**
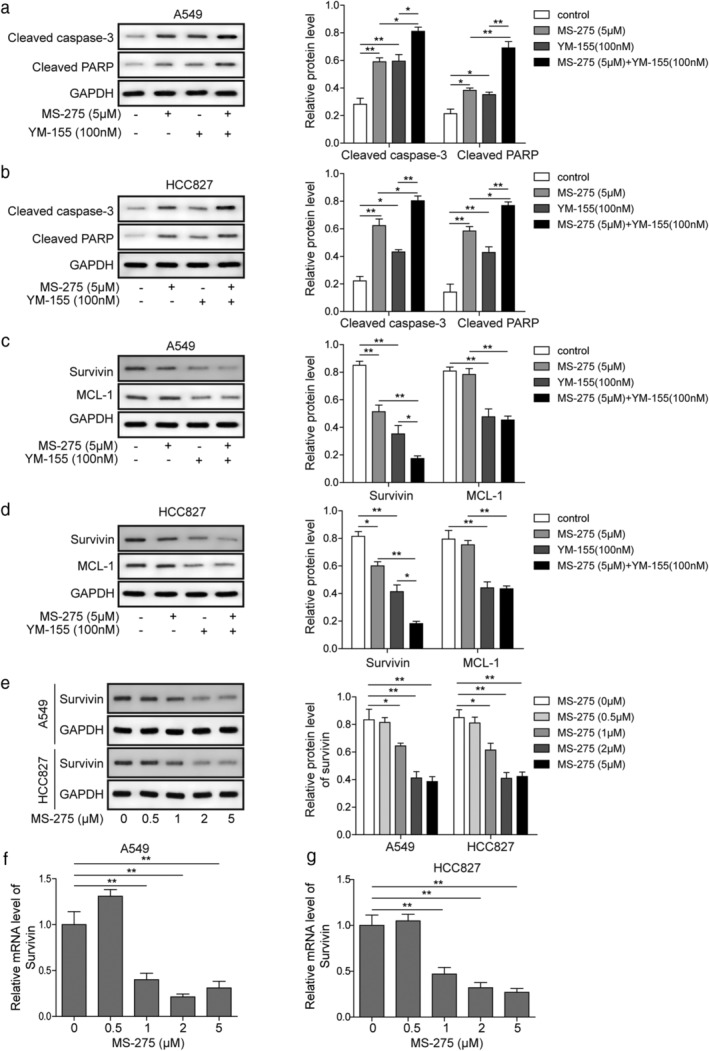
Combination treatment enhances the inhibition effect of YM‐155 on survivin and the proapoptotic effect of YM‐155. (**a,b**) Western blots of the cleaved capased‐3 and cleaved PARP levels in A549 and HCC827 following treatment with 5 μM MS‐275, 100 nM YM‐155, or both for 48 hours. (**c,d**) Western blot of survivin and MCL‐1 levels in A549 and HCC827 following treatment with 5 μM MS‐275, 100 nM YM‐155, or both for 48 hours. (**e**) Western blot and (**f,g**) quantitative PCR of survivin levels in A549 and HCC827 following treatment with indicated concentrations of MS‐275. *P < 0.05, **P < 0.01. Data are presented as mean ± standard deviation. At least three independent experiments were performed. GAPDH, glyceraldehyde 3‐phosphate dehydrogenase.

Cells were treated with both MS‐275 and YM155 and cell viability was examined by MTT assay (Fig 2a,b). YM‐155 inhibited cell proliferation in a dose‐dependent manner. The combination of 5 μM MS‐275 and 100 nM YM‐155 showed a significant inhibitory effect on the proliferation of A549 (72.81 ± 2.82) and HCC827 (58.88 ± 4.24), with more than 27% and 41% inhibition, respectively, while 2 μM MS‐275 showed slight enhancement but no statistical significance. The LUAD cells administered with MS‐275, YM155, or both and colony formation were examined using agar colony formation assay (Fig 2c–e). Individual treatment of 5 μM MS‐275 or 100 nM YM‐155 reduced colony events in A549 cells and HCC287 cells. Meanwhile the combination of MS‐275 and YM‐155 treatment further reduced the colony numbers of A549 (115.5 ± 17.70 vs. 61.5 ± 7.18 YM‐155 alone) and HCC287 cells (87.5 ± 11.35 vs. 45 ± 7.12 YM‐155 alone) compared to single treatment with YM155. Furthermore, in vivo xenograft tumorigenesis assay revealed that the combination of 20 mg/kgMS‐275 and 5 mg/kgYM155 induced significant inhibition of tumor growth 30 days after the xenograft. Therefore, the combination of YM155 and MS‐275 exhibited a significant synergistic effect on the proliferation (Fig 3f) and tumorigenesis (Fig 3g) of LUAD cells compared to that induced by the administration of YM‐155 alone.

### Combination treatment enhances the inhibition effect on survivin and the proapoptosis effect of YM‐155

We studied the expression level of proapoptotic proteins in LUAD cells by Western blot analysis. The cleaved capased‐3 and cleaved PARP levels were low in the control (Fig 3a,b). After treatment with YM‐155, MS‐275, or a combination, the expression levels of the indicated proteins were significantly enhanced in A549 and HCC827 cell lines. Moreover, our results demonstrated that combination treatment further increased the levels of the proapoptotic proteins compared to treatment with MS‐275 or YM155 alone. Furthermore, we investigated whether YM155, MS‐275, or both could inhibit survivin expression in human lung cell lines. We found that 5 μM MS‐275 can significantly suppress survivin expression but not MCL‐1 expression. In contrast, 100 nM YM155 significantly suppressed survivin and MCL‐1 expression. Meanwhile, the combination of YM155 and MS‐275 treatment could further reduce the expression of survivin but not MCL‐1 (Fig 3c,d). Survivin expression was significantly suppressed by treatment with 2μM or 5μM MS‐275 alone (Fig 3e–g).

### MS‐275 reduces miR‐138 and miR‐195 methylation levels by upregulating acetylated H3 and decreasing DNA methyltransferase expression

**Figure 4:**
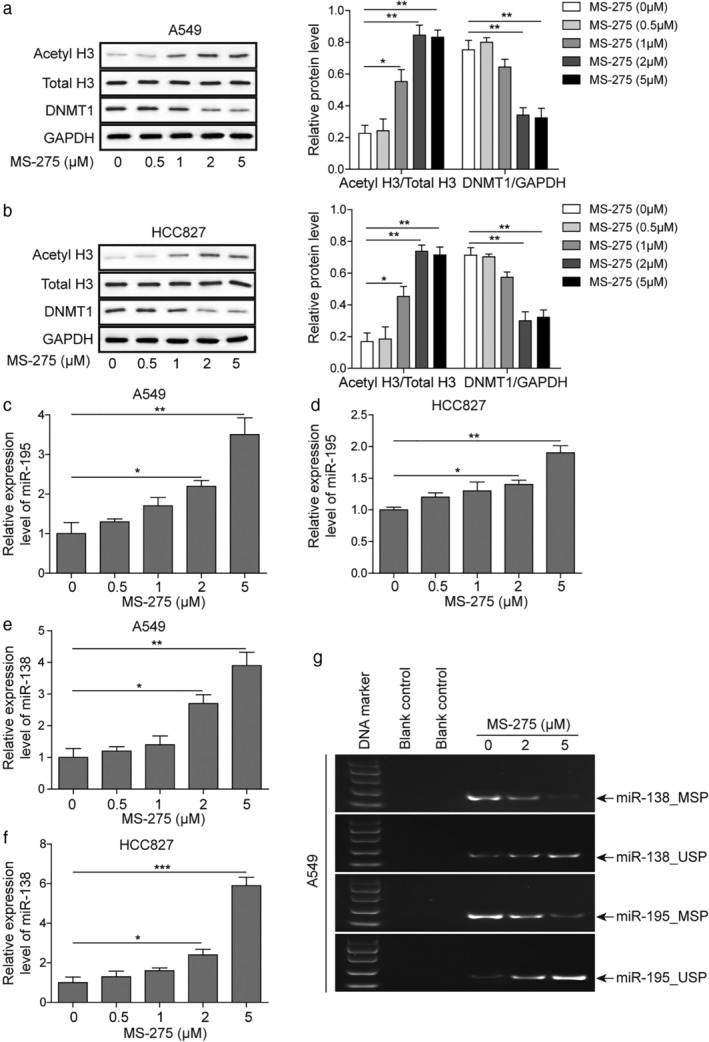
MS‐275 reduces the methylation level of miR‐138 or miR‐195 by upregulating acetylated H3 and decreasing the expression of *DNMT1*. (**a,b**) Western blot of acetylated histone H3/total histone H3 and DNMT1 levels in A549 and HCC827 following treatment with different concentrations of MS‐275. (**c–f**) Quantitative PCR of miR195 and miR138 levels in A549 and HCC827 following treatment with 5 μM MS‐275. *P < 0.05, **P < 0.01, ***P < 0.001. (**g**) Methylation‐specific PCR analysis of miR‐138 and miR‐195 following treatment with MS‐275. Data are presented as mean ± standard deviation. At least three independent experiments were performed. GAPDH, glyceraldehyde 3‐phosphate dehydrogenase.

Many tumor suppressive miRNAs are inhibited as a result of hypermethylation by DNMTs.^19^ To investigate the mechanism of the synergistic effect of MS‐275, we detected the expression or modification of miRNAs and acetylated histone H3/total histone H3. MS‐275 is a highly effective HDAC inhibitor. The level of acetyl H3 increased in a dose dependent manner with constant total H3, while DNMT1 expression was decreased with increased concentrations of MS‐275 (Fig 4a,b). Further, we measured the relative expression levels of miR‐195 and miR‐138 in LUAD cells by quantitative PCR. The expression levels of miR195 and miR138 increased in a dose dependent manner (Fig 4c–f). Meanwhile, treatment with 2 and 5 μM MS‐275 for 48 hours significantly reduced the methylation levels of miR‐138 and miR‐195 (Fig 4g). These results suggest that MS‐275 reduces the methylation of miR‐138 and miR‐195 to elevate the expression of these miRNAs by inhibiting DNMT1 expression.

### Inhibition effect of miR‐138 or miR‐195 on LUAD proliferation combined with or without YM‐155

**Figure 5:**
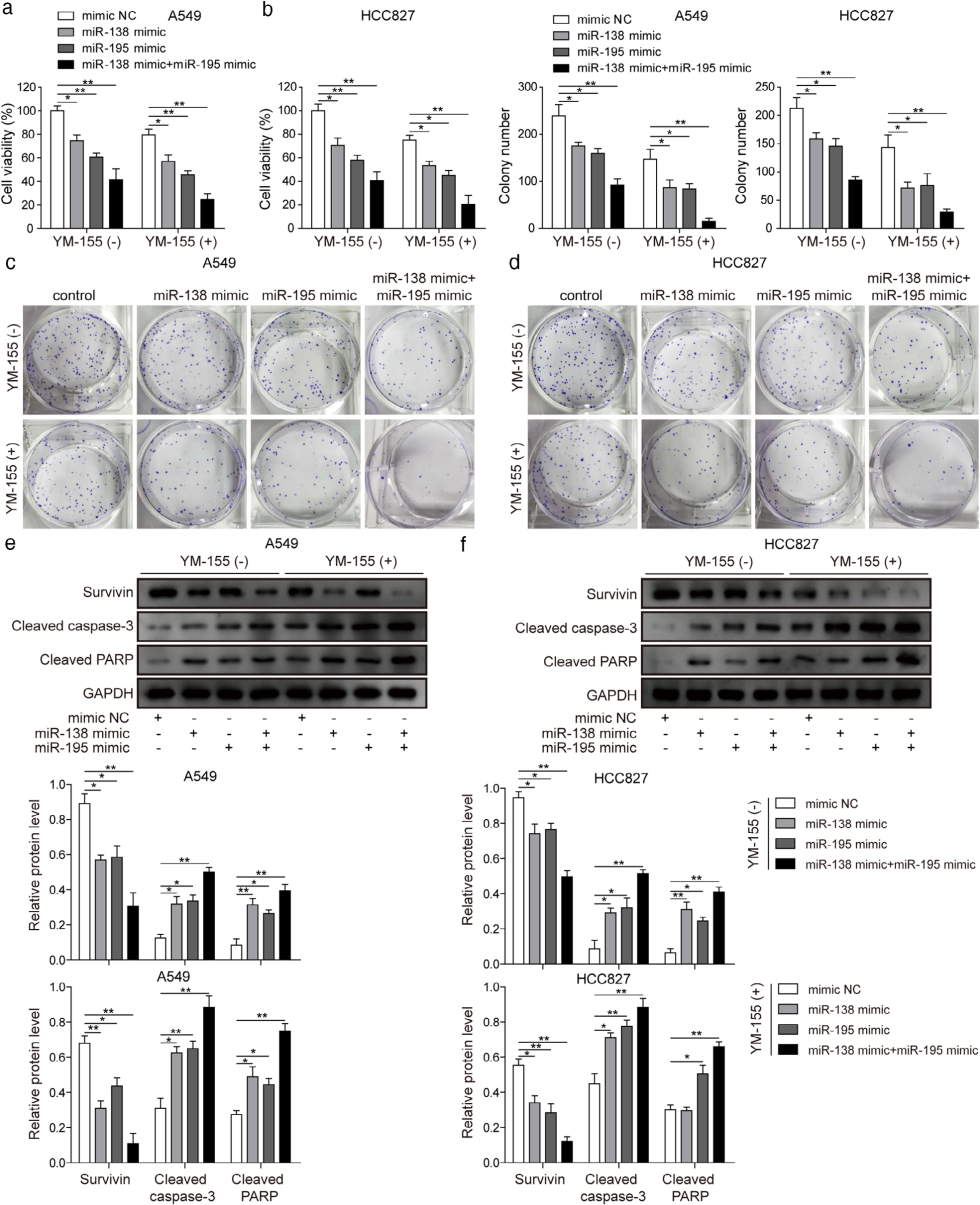
The inhibition effect of miR‐138 or miR‐195 on lung adenocarcinoma (LUAD) proliferation and the synergizing effect with YM‐155. (**a,b**) Methyl thiazolyl tetrazolium and (**c,d**) colony formation assay results of LUAD cell lines A549 and HCC827 following treatment with the negative control (NC), miR‐138, and miR‐195 mimics plus 100 nM YM‐155. (**e,f**) Western blot of survivin, cleaved capased‐3, and cleaved PARP levels in A549 and HCC827 following treatment with NC, miR‐138, and miR‐195 mimics plus 100nM YM‐155. *P < 0.05, **P < 0.01. Data are presented as mean ± standard deviation. At least three independent experiments were performed. GAPDH, glyceraldehyde 3‐phosphate dehydrogenase.

It has recently been reported that miR‐138 and miR‐195 function as tumor suppressors.^20^, ^21^ To investigate the synergistic activity of miR‐138 or miR‐195 with YM‐155 in LUAD cells, we examined the cytotoxic efficacy of YM‐155 with or without miRNAs in a panel of LUAD cell lines. After 48 hours of treatment, cell viability was assessed by MTT assay. Cell viability decreased significantly in cells treated with miR‐138 mimic (74.39 ± 4.96 for A549, 70.37 ± 6.36 for HCC827), miR‐195 (60.41 ± 3.53 for A549, 58.07 ± 4.24 for HCC827) mimic, or both (41.42 ± 9.19 for A549, 40.61 ± 7.78 for HCC827) without YM‐155 compared to the control group. After treatment with miR‐138 (57.02 ± 5.65 for A549, 53.59 ± 3.53 for HCC827), miR‐195 (45.51 ± 3.53 for A549, 45.34 ± 4.24 for HCC827), or both (24.37 ± 4.95 for A549, 20.58 ± 7.47 for HCC827) plus YM‐155, the cell viability further reduced (Fig 5a,b) compared to YM‐155 alone (79.65 ± 4.95 for A549, 75.02 ± 4.24 for HCC827). Meanwhile miR‐138, miR‐195, or both significantly inhibited the colony numbers of A549 and HCC827 cell lines and miR‐138 (86.5 ± 16.25 for A549, 71.5 ± 10.61 for HCC827), miR‐195 (84 ± 11.31 for A549, 76.5 ± 20.51 for HCC827), or both (15.5 ± 6.36 for A549, 29 ± 5.65 for HCC827) enhanced the inhibition efficacy of YM‐155 compared to YM‐155 alone (147 ± 21.21 for A549, 143.5 ± 21.92 for HCC827) (Fig 5c,d). To investigate the mechanisms underlying the synergistic effect, the expression of survivin, cleaved capased‐3, and cleaved PARP was detected. We found that survivin was downregulated while cleaved capased‐3 and cleaved PARP were elevated by miR‐138, miR‐195, or both, and the results also showed that YM‐155 further reduced the expression of survivin and increased the expression of cleaved capased‐3 and cleaved PARP (Fig 5e,f).

### MiR‐138 and miR‐195 inhibit the expression of survivin by directly binding to the 3’ untranslated region

**Figure 6:**
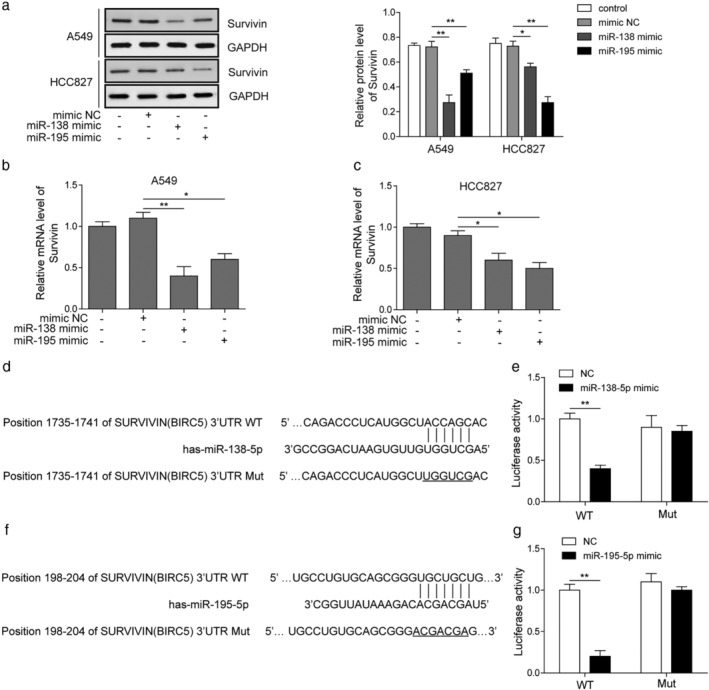
MiR‐138 and miR‐195 inhibit the expression of survivin by directly binding to 3‘ untranslated region. (**a**) Western blot and (**b,c**) quantitative PCR of survivin levels in A549 and HCC827 following treatment with negative control (NC), miR‐138, and miR‐195 mimics. (**d,f**) Illustration of the putative binding site between miR‐139 or miR‐195 and survivin. (**e,g**) Luciferase reporter assay of survivin levels in A549 and HCC827 following treatment with NC, miR‐138, and miR‐195 mimics. *P < 0.01. Data are presented as mean ± standard deviation. At least three independent experiments were performed. GAPDH, glyceraldehyde 3‐phosphate dehydrogenase, WT, wild‐type.

In view of their repressive effects on lung cancer growth, we used TargetScan to predict the targets of miR‐138 and miR‐195 and found that survivin was a putative target of both miR‐138 and miR‐195. We investigated the expression level of survivin by Western blot and quantitative PCR analysis to determine whether miR‐138 and miR‐195 could inhibit the expression of survivin independent of YM‐155. The endogenous protein expression of survivin dramatically decreased in A549 and HCC827 cells, and miR‐138 or miR‐195 also significantly inhibited survivin messenger RNA expression (Fig 6a–c). To investigate the mechanisms underlying the inhibition effect, we employed a luciferase reporter system including the survivin 3’UTR (Luc‐survivin) containing wild type or presumed mutated miR‐138 and miR‐195 target sites (Fig 6d,f). MiR‐138 and miR‐195 significantly weakened the luciferase activity of Luc‐survivin in the wild type group, whereas luciferase reporter activity in the mutational target site was not influenced by the miRNA mimic in A549 or HCC827 cell lines (Fig 6e,g). These results suggest that survivin is directly targeted and inhibited by miR‐138 and miR‐195 in LUAD.

### The inhibition effect of MS‐275 on non‐small cell lung cancer proliferation is dependent on miR‐138 and miR‐195

**Figure 7:**
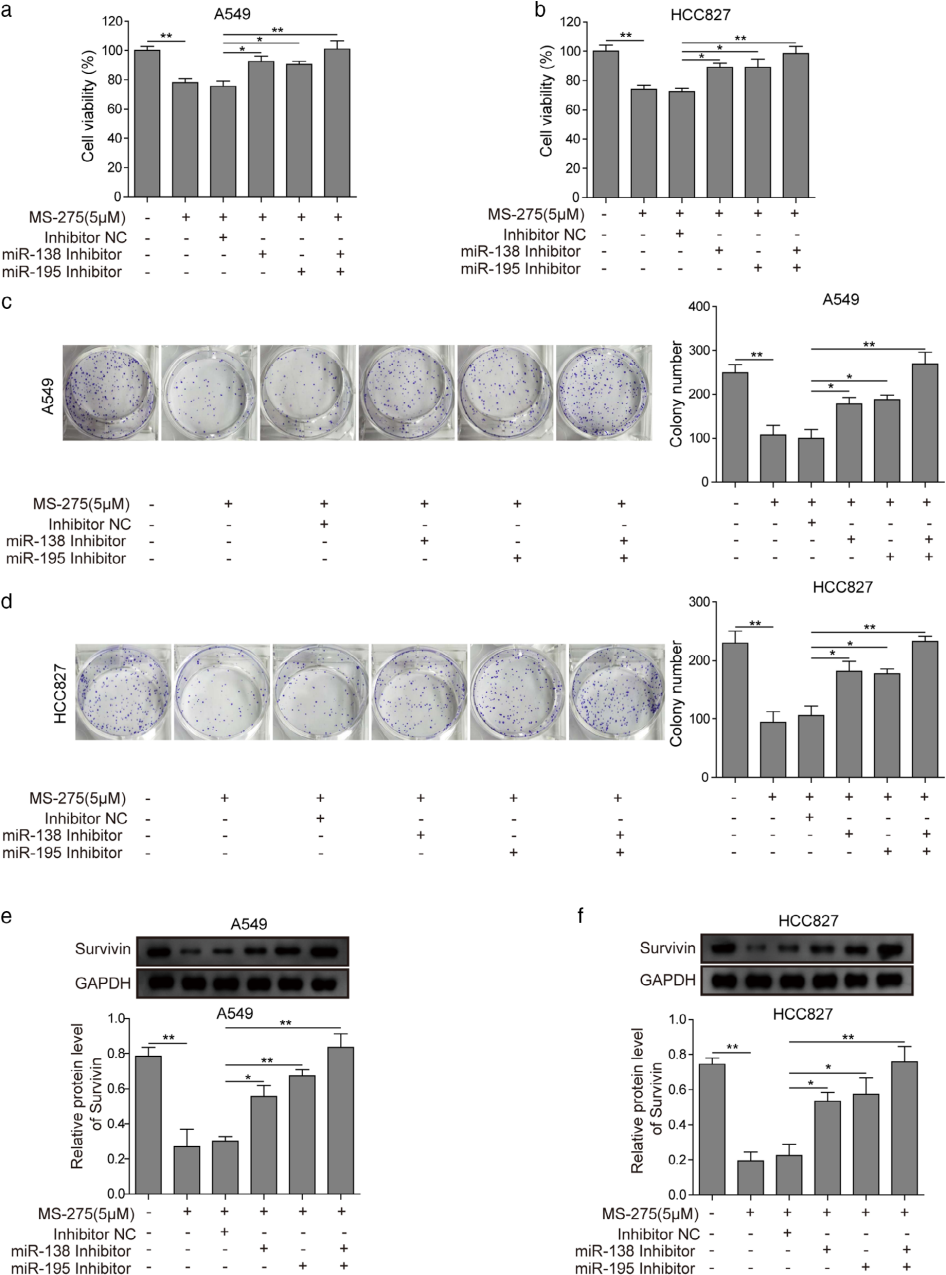
The inhibition effect of MS‐275 on non‐small cell lung cancer proliferation is dependent on miR‐138 and miR‐195. (**a,b**) Methyl thiazolyl tetrazolium, (MTT) and (**c,d**) colony formation assay results of lung adenocarcinoma (LUAD) cell lines A549 and HCC827 following treatment with the negative control (NC), miR‐138, or miR‐195 inhibitors plus 5 μM MS‐275. (**e,f**) Western blots of survivin levels in A549 and HCC827 following treatment with the NC, miR‐138, or miR‐195 inhibitors plus 5 μM MS‐275. *P < 0.05, **P < 0.01. Data are presented as mean ± standard deviation. At least three independent experiments were performed.

To investigate whether the inhibition effect of MS‐275 on NSCLC proliferation is dependent on miR‐138 and miR‐195, we used the miRNA inhibitor to silence miR‐138 or miR‐195 when treated with MS‐275. The results of MTT assay showed that the addition of the inhibitor alone or both can reverse the inhibition of 5μM MS‐275 on NSCLC proliferation (Fig 7a,b). Furthermore, the results of colony formation also showed that the inhibition of 5μM MS‐275 on the formation of clones was offset, regardless of whether the miRNA inhibitor alone or both were added (Fig 7c,d). When adding MS‐275 and the miRNA inhibitor at the same time, the inhibition of survivin by MS‐275 was restored when miRNA inhibitors alone or both were added (Fig 7e,f).

## Discussion

The key finding of our study is that MS‐275 increases the inhibitory effect of YM‐155 on LUAD proliferation. The combination of YM155 and MS‐275 induces a significant antitumor effect in lung carcinoma compared to that induced by YM‐155 or MS‐275 alone. This synergistic effect contributes to the suppression effect of HDAC caused by MS‐275, which increases the level of acetylation H3 and then inhibits DNMT expression to the demethylation promoter of miR‐138 and miR‐195, which can target survivin to induce the apoptosis of A549 and HCC827 cell lines. These results demonstrate that the combination of MS‐275 and YM‐155 may play a potential role in the treatment of LUAD.

YM‐155, a member of the IAP family, inhibits survivin expression. Since its discovery in a high‐throughput survivin gene promoter assay, the anticancer activity of YM155 has been reported in a wide variety of mouse tumor xenograft models.^22^, ^23^ YM‐155 shows synergistic antitumor activities with IL‐2 in renal cell carcinoma,^24^ and with ABT‐737 in human glioma cells.^25^ Previous reports have demonstrated that combination treatment can enhance the antitumor effect of YM‐155. MS‐275, also called entinostat, is a benzamide‐type HDAC inhibitor specific to class I HDACs. The antiproliferative effects of MS‐275 have been shown in several cancer cell lines, including prostate,^26^ bladder,^27^ and pancreatic cancers.^28^ The combination of phosphodiesterase inhibitor and MS‐275 is reported to enhance anticancer activity in human breast cancer.^29^ Meanwhile a phase I–II study including 45 pretreated patients with metastatic or recurrent NSCLC tested the successful combination of azacitidine and MS‐275 in preclinical models.^30^ In our study, the effects of the combination therapy of YM‐155 and MS‐275 were evaluated in A549 and HCC287 cell lines. The combination of MS‐275 and YM‐155 treatment significantly reduced the colony formation of A549 and HCC287 cell lines compared to treatment with MS‐275 or YM155 alone. This is first time the synergistic effect of MS‐275 and YM‐155 has been reported, and suggests a new direction for the future application of YM‐155. Whether this combination therapy is effective for other various cancers needs to be confirmed. There are also some shortcomings of concern, such as more severe side effects^31^ and difficulties in clinical application.

Some forms of epigenetic modification have potential application in oncology therapy, such as DNA methylation of noncoding miRNA expression.^32^ *DNMT1* is considered the principal cause maintaining these epigenetic modifications.^33^ Several previous studies have shown that both histone deacetylation and DNA methylation can silence genes, including some tumor suppressor genes, which suggests that it is important to develop DNMT and HDAC inhibitors.^34^ Juergens *et al.* reported that DNMT gene inhibitors show potential for treating solid tumors, including NSCLC.^30^ MS‐275 has shown promising results in solid tumors in recent clinical trials.^35^ Meanwhile, HDAC inhibitors can downregulate the expression of DNMT1‐mediating multiple myeloma cell proliferation and DNMT1 expression is regulated by c‐Myc, the degradation of which is triggered by HDAC inhibition.^17^ In the present study, MS‐275 inhibited DNMT1 expression by upregulating H3 acetylation, consistent with the findings of previous reports.^17^ Moreover, for the first time, we also showed that MS‐275 inhibits DNMT1 and further reduces the methylation of miR‐195 and miR‐138 genes.

Many studies have indicated that miRNAs play important roles in the biogenesis and regulatory machinery of NSCLC.^36^ The genetic regulation of miRNAs is similar to the regulation of messenger RNAs, and the process regulators include specific transcription factors or proteins that usually interact with the promoter.^37^ For example, miR‐124a has been reported to own a CpG island near its promoter region.^19^ In normal cells, DNA methylation does not occur. In lung cancer, DNA methylation may occur throughout the genome with both low and high levels of methylation of the gene promoter region.^38^ It has also been reported that hypermethylation of miRNAs in the CpG islands lead to downregulation of miRNAs.^39^ In the present study, we found that MS‐275 can increase the expression of miR‐138 and miR‐195 as a result of the reduced methylation of these miRNAs. Recently miR‐195 has been reported as a tumor suppressor in various cancers, including NSCLC, as it targets survivin.^40^ In addition, recent studies have shown that miR‐138 is frequently downregulated in lung cancer cell lines. Some reports have revealed that miR‐138 could target PDK1,^21^ CCND3,^41^ and the enhancer of zeste homolog 2.^42^ Our results demonstrate that miR‐138 and miR‐195 target survivin in NSCLC and enhance the tumor suppressive effect of YM‐155. This indicates that miR‐138 and miR‐195 upregulation induced by MS‐275 may also play a tumor‐suppressing role by inhibiting other targets. However, other targets of miR‐138 and miR‐195 may be involved in this synergistic effect. Our results show that YM‐155 plus MS‐275 has a more significant inhibitive effect via the miRNA‐mediated targeting of survivin, while the synergic effect is more likely the result of the independent repression of survivin, which may restrain the application in future. Hence, we conclude that the combination effect is more likely enhancing rather than synergetic. However, the revealed mechanism sheds new light on survivin upregulation in NSCLC, specifically in patients with increased DNMT1 and/or survivin levels.

In summary, we have shown that the combination of YM‐155 and MS‐275 potently inhibits the proliferation of LUAD cells and is associated with reduced DNMT1 expression to elevate miR‐138 and miR‐195 expression, which targets survivin. Our results show that the antitumor effect of YM‐155 and MS‐275 is correlated with the methylation inhibition of miRNA status, which may provide a new direction for the future application of YM‐155.

## Disclosure

No authors report any conflict of interest.
